# Integrating Body Composition and Nutritional Indices: A Novel Prognostic Tool for Survival in Pancreatic Cancer

**DOI:** 10.1002/jcsm.70006

**Published:** 2025-07-09

**Authors:** Yiting Xu, Yang Chen, Gaowei Jin, Chenrui Yao, Yangyang Wang, Ziyang Wei, Zhihang Cai, Xuanhao Gu, Binbin Deng, Peilu Wang, Yuxiong Feng, Qi Zhang, Tingbo Liang

**Affiliations:** ^1^ Department of Hepatobiliary and Pancreatic Surgery, The First Affiliated Hospital Zhejiang University School of Medicine Hangzhou China; ^2^ Zhejiang Provincial Key Laboratory of Pancreatic Disease, The First Affiliated Hospital Zhejiang University School of Medicine Hangzhou China; ^3^ Department of Gastroenterology The First Affiliated Hospital of Wenzhou Medical University Wenzhou China; ^4^ Department of Rehabilitation Medicine The First Affiliated Hospital of Wenzhou Medical University Zhejiang China; ^5^ Department of Nutrition and Food Hygiene, School of Public Health, Institute of Nutrition Fudan University Shanghai China; ^6^ Zhejiang key Laboratory of Pancreatic Disease, The First Affiliated Hospital, Zhejiang key Laboratory of Frontier Medical Research on Cancer Metabolism, Institute of Translational Medicine Zhejiang University School of Medicine Hangzhou China; ^7^ Institute of Fundamental and Transdisciplinary Research, Cancer Center Zhejiang University Hangzhou China; ^8^ The Innovation Center for the Study of Pancreatic Diseases of Zhejiang Province Hangzhou China; ^9^ MOE Joint International Research Laboratory of Pancreatic Diseases, The First Affiliated Hospital Zhejiang University School of Medicine Hangzhou China; ^10^ Zhejiang University Cancer Center Hangzhou China

**Keywords:** body composition, nutrition, pancreatic cancer, prognosis, survival

## Abstract

**Background:**

Sarcopenia and malnutrition have been independently associated with a poorer prognosis in pancreatic ductal adenocarcinoma (PDAC), but their combined association with patient outcomes is not fully understood. This study aimed to systematically evaluate the synergistic effects of body composition parameters and nutritional index as prognostic indicators in patients with PDAC.

**Methods:**

A total of 596 patients with PDAC who underwent surgical resection from two centres were initially enrolled in this retrospective study. Body composition parameters, including the skeletal muscle index (SMI), subcutaneous adipose tissue index (SATI), visceral adipose tissue index (VATI) and skeletal muscle density (SMD), were assessed using a single cross‐sectional image at the L3 level from preoperative computed tomography scans. The prognostic nutritional index (PNI) was used to assess nutritional status. The combined indices were defined as body composition parameters multiplied by PNI.

**Results:**

A total of 463 patients were finally included in the analysis, with 339 in the training cohort and 124 in the validation cohort. The median (interquartile) age was 66 (60–72) years, and 274 (59.2%) were male. The median values of SMI, SATI, VATI, SMD, as well as the combined indices of these parameters with PNI, varied significantly by sex in the training cohort. Patients were categorized into sex‐specific quartiles (Q1 to Q4) based on SMI × PNI levels. Both overall survival (OS) and disease‐free survival (DFS) exhibited significant differences across these quartiles (*p* < 0.001). Though all body composition parameters and their combinations with PNI were independent predictors of OS in multivariate analysis, the combination of SMI × PNI demonstrated superior prognostic performance compared to other indices (c‐statistics: 0.767, AICc: 1648.8). These results remained consistent across stratified analysis. The external validation cohort confirmed that SMI × PNI exhibited enhanced predictive and discriminative power compared with other indices.

**Conclusions:**

SMI × PNI represents a robust and accessible prognostic tool for assessing survival in patients with PDAC. Further prospective studies are needed to validate its effectiveness across diverse populations and clinical settings.

## 1 Introduction

Despite significant advancements in surgical and medical therapies, the survival rate of pancreatic ductal adenocarcinoma (PDAC) remains the lowest among solid tumours, with a 5‐year survival rate of < 10% [1]. Complete surgical resection is currently the only curative option, but fewer than 20% of patients present with localized and potentially curable tumours at diagnosis [2]. What is more concerning is that some patients still have a poor prognosis even after surgical resection. Therefore, exploring prognostic factors for patients with PDAC who have undergone radical resection is of great significance.

Body composition, including adipose and muscle tissues, can be assessed using body mass index (BMI) or more precisely through parameters, such as skeletal muscle volume, visceral adipose tissue (VAT), subcutaneous adipose tissue (SAT) and skeletal muscle density (SMD) measured from computed tomography (CT) images at the level of the third lumbar vertebra [3]. Increasing evidence suggests that body composition parameters may predict prognosis in various cancers [4, 5]. Sarcopenia, defined as the degenerative loss of skeletal muscle mass, was associated with poor clinical outcomes in patients undergoing surgery for hepatocellular carcinoma, colorectal cancer and pancreatic cancer [4, 5, 6]. In addition, Han et al. found that SAT and VAT showed different effects on gastric cancer patients with cachexia, and the loss of SAT was more pronounced than that of VAT [7]. Besides, body composition parameters have also been shown to be related to postoperative complications and adverse events across a variety of surgical settings. For instance, Shahab Hajibandeh and colleagues found that sarcopenia was associated with a higher 30‐day mortality rate in patients undergoing emergency laparotomy [8]. Moreover, a 10‐year multicentre study revealed that sarcopenic obesity—coexisting sarcopenia and obesity—was associated with higher postoperative complication rates after hepatectomy compared to sarcopenia alone [9]. These findings highlight the predictive value of body composition analysis in surgical oncology, suggesting its potential role in optimizing preoperative risk assessment and postoperative care.

Meanwhile, nutritional assessment indicators, including the Global Leadership Initiative on Malnutrition (GLIM), the controlling nutritional status (CONUT) and the prognostic nutritional index (PNI), have been closely linked to outcomes in patients with cancer [10, 11, 12]. Among these, the PNI, derived from routine biochemical measures, provides a simple and objective tool for clinical evaluation. Notably, accumulating evidence indicated that the PNI serves as an independent prognostic factor across multiple cancer types [12]. Previous studies have shown that combining body composition parameters with nutritional assessment may provide a more comprehensive evaluation of patient prognosis and postoperative adverse events. For example, Shinya Abe et al. developed a score combining PNI and psoas muscle mass index, which was strongly associated with prognosis in patients with locally advanced rectal cancer [13]. Additionally, malnutrition and sarcopenia in cirrhosis have been linked to adverse post‐transplant outcomes, particularly infections and prolonged hospitalization [14]. However, these synergetic effects have yet to be fully explored in the context of PDAC.

Interestingly, systemic inflammation is another potential factor in predicting outcomes for patients with PDAC. Inflammatory proteins such as C‐reactive protein (CRP), interleukin‐6 (IL‐6) and tumour necrosis factor (TNF‐α) have been associated with PDAC progression [15, 16, 17]. These cytokines not only influenced muscle homeostasis and the regulation of muscle protein turnover, but also played a vital role in the development of malnutrition [18, 19]. Therefore, it is plausible that muscle mass and nutrient metabolism may intersect through common pathways to affect survival outcomes. Their synergistic effects in patients with PDAC need to be further studied.

Herein, we retrospectively analysed the prognostic significance of body composition parameters and their integration with nutritional assessment in patients with PDAC undergoing surgery from two centres. We divided patients into sex‐specific quartiles (Q1 to Q4) according to the SMI × PNI levels and achieved great performance for prognostic stratification, which may contribute to the advancement of precision medicine.

## 2 Methods

### 2.1 Study Population

**FIGURE 1:**
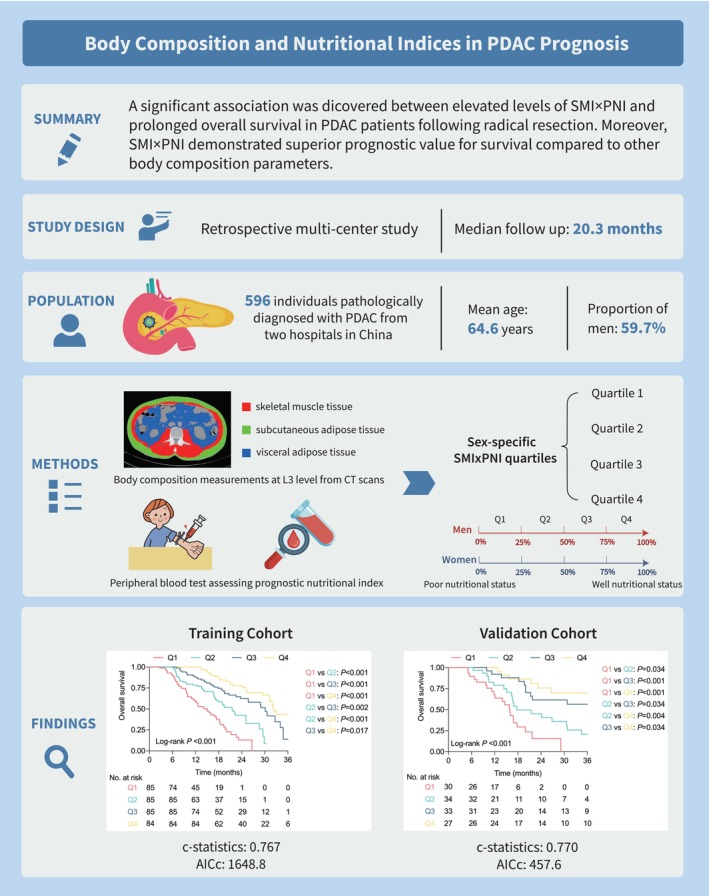
Graphical Abstract. Body Composition and Nutritional Indices in PDAC prognosis.

A graphical abstract was provided to elucidate the association between body composition, nutritional indices and PDAC prognosis (Figure 1). This multicentre retrospective study enrolled patients with pathologically confirmed PDAC who underwent surgical resection at two hospitals in China: the First Affiliated Hospital, Zhejiang University School of Medicine (FAHZU) (*n* = 433) and the First Affiliated Hospital of Wenzhou Medical University (*n* = 163). Patients at FAHZU between Jan. 2021 and Jan. 2023 were allocated to the training cohort, while patients at Wenzhou centre between Jan. 2018 and Jan. 2023 comprised the external validation cohort. This study was conducted in accordance with the Helsinki Declaration and was approved by the Ethics Committee of FAHZU and the First Affiliated Hospital of Wenzhou Medical University. The requirement for informed consent was waived due to the retrospective nature of the study.

**FIGURE 2:**
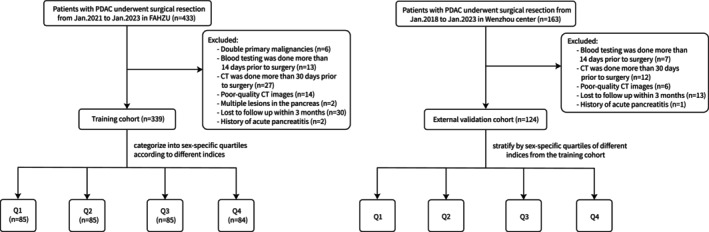
The flowchart of our study.

The inclusion criteria were (1) pathologically and histologically confirmed PDAC; (2) patients who underwent radical surgical resection, including locally advanced/borderline resectable tumours receiving neoadjuvant therapy, and selected metastatic cases achieving remission; (3) blood testing performed within 14 days prior to surgery, including serum albumin and lymphocyte count measurements; and (4) underwent abdominal‐pelvic computed tomography (CT) within 30 days prior to surgery. The exclusion criteria were (1) patients with double primary malignancies; (2) multiple lesions in the pancreas; (3) loss to follow‐up within 3 months after surgery; (4) a history of acute pancreatitis. The flow chart of our study is presented in Figure 2.

### 2.2 Clinical Data Assessment

Clinical data from electronic medical records included demographic information (age, sex, body mass index [BMI], hypertension, diabetes, whether received neoadjuvant or adjuvant therapy), laboratory data (neutrophil count, lymphocyte count, haemoglobin, cholesterol, low density lipoprotein [LDL], high density lipoprotein [HDL], albumin, carcinoembryonic antigen [CEA] and carbohydrate antigen 19‐9 [CA19‐9] levels), and pathological data (tumour location, maximal tumour size, pathological TNM stage and tumour differentiation). Obesity was defined as BMI ≥ 25 kg/m^2^ based on the WHO Western Pacific Region criteria for obesity in Asian populations [20]. PNI was calculated using the formula: PNI = albumin (g/L) + 5* lymphocyte count (x10^9/L) [21].

### 2.3 Measurements of Body Composition Parameters

**FIGURE 3:**
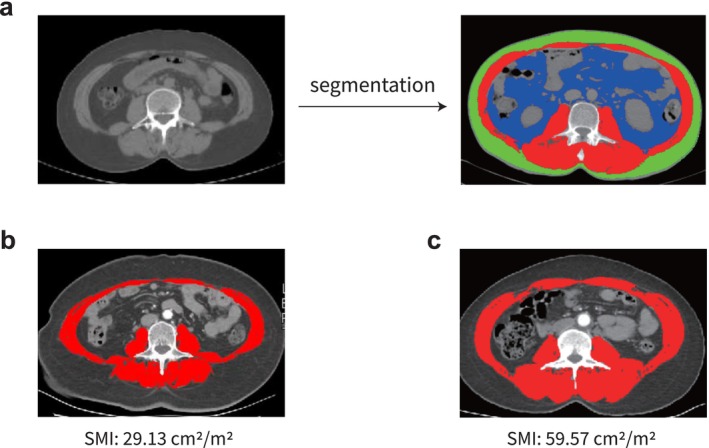
Measurements of body composition parameters. The skeletal muscle volume area (SMA), subcutaneous adipose tissue area (SATA), and visceral subcutaneous adipose tissue area (VATA) were evaluated from a single image at the third lumber vertebra (L3). Analysed CT scans was obtained within 30 days before surgery. The different abdominal tissues were segmented according to their characteristic radiodensity ranges. Skeletal muscle index (SMI), subcutaneous adipose tissue index (SATI), and visceral adipose tissue index (VATI) was calculated by dividing by the square of the patient's height (m^2^). (a) The SMA, SATA and VATA are displayed in red, green and blue, respectively. (b) Example: A 72‐year‐old woman with less skeletal muscle tissue. (c) Example: A 56‐year‐old man with more skeletal muscle tissue.

All study patients underwent a preoperative abdominal CT scan within 30 days of surgery. The segmentation of the different abdominal tissues was performed semi‐automatically using the analysis software SliceOMatic V5.0 (Tomovision) based on characteristic radiodensity ranges (Figure 3). The skeletal muscle volume area (SMA), subcutaneous adipose tissue area (SATA) and visceral subcutaneous adipose tissue area (VATA) were evaluated from a single image at the third lumbar vertebra (L3) using Hounsfield unit thresholds of −29 to +150 for skeletal muscle, −190 to −30 for subcutaneous adipose tissue and −150 to −50 for visceral subcutaneous adipose tissue, respectively [22]. The skeletal muscle density (SMD) was assessed by estimating the mean HU value of the SMA. To account for variations in patient height, each area was normalized by dividing by the square of the patient's height (m^2^), yielding the skeletal muscle index (SMI), subcutaneous adipose tissue index (SATI) and visceral adipose tissue index (VATI), respectively. The formulas for calculating these indices are as follows: SMI (cm^2^/m^2^) = SMA/height^2^, SATI (cm^2^/m^2^) = SATA/height^2^, VATI (cm^2^/m^2^) = VATA/height^2^.

The combined indices were calculated using the following formula: SMI × PNI = SMI (HU) × PNI; SATI×PNI = SATI (HU) × PNI; VATI×PNI = VATI (HU) × PNI; SMD × PNI = SMD (HU) × PNI.

### 2.4 Statistical Analysis

For continuous variables of clinicopathological characteristics, the unpaired Student's *t* test was used for those with normal distribution, the Wilcoxon rank sum test or Kruskal–Wallis test was applied for abnormal distribution data, and the Chi‐square test or Fisher's exact test was used for the dichotomous variables. Normally distributed continuous variables were expressed as the mean ± standard deviation (SD), whereas non‐normally distributed continuous variables were expressed as median and interquartile range (IQR). Categorical variables were presented as percentages (%).

Overall survival (OS) was defined as the interval between the date of surgery and the date of death or last follow‐up. Disease‐free survival (DFS) was defined as the period from the time of surgery to the first confirmed recurrence or death. OS and DFS were estimated using the Kaplan–Meier method and compared between groups using the log‐rank test. Clinicopathological variables identified as potentially significant risk factors (*p* < 0.05) in univariate analysis were included in the multivariate Cox regression analysis. To compare the prognostic capabilities of different indices, Harrell's c‐statistics and the corrected Akaike information criterion (AICc) were calculated. All statistical analyses were performed using the SPSS v27.0 (IBM, Armonk, NY), R version 4.2.2, and Prism 10.0 (GraphPad Soft Inc., San Diego, CA) software programs. A two‐tailed *p* value of < 0.05 was considered statistically significant.

## 3 Results

### 3.1 Baseline Characteristics

A total of 463 patients were included in the analysis, with 339 patients in the training cohort and 124 patients in the external validation cohort, respectively (Table S1). Of the 339 participants in the training set, the average age of patients was 67 years, and 59.0% were male. The baseline characteristics of patients in our cohort are presented in Table S2. The median values of SMI, SATI, VATI and SMD × varied significantly by sex (Figure S1 and Figure S2). Similarly, significant differences were observed in SMI × PNI, SATI × PNI, VATI × PNI and SMD × PNI across the sex‐specific groups. Hence, we categorized study cohorts into sex‐specific quartiles (Q1 to Q4) based on SMI × PNI levels, and the male and female subgroups were combined to create four new groups comprising 85, 85, 85 and 84 participants, respectively.

The baseline clinical and pathological characteristics of the study patients stratified by quartiles of SMI × PNI levels are shown in Table 1. With increasing SMI × PNI levels, patients tended to be younger and exhibited higher levels of lymphocyte count, haemoglobin, cholesterol, LDL, PNI, SMI, SATI, VATI and SMD along with a lower proportion of pancreatic head cancer (*p* < 0.05). Notably, AJCC IV (M1) cases (*n* = 22, 6.5%) were distributed across quartiles (Q1: 4; Q2: 7; Q3: 8; Q4: 3, *p* = 0.355), all of whom underwent radical resection following sustained metastasis remission (RECIST 1.1) with gemcitabine/nab‐paclitaxel‐based or FOLFIRINOX‐based neoadjuvant therapy (at least 4 cycles).

**TABLE 1:** Patient characteristics according to sex‐specific quartiles of SMI × PNI levels.

	Q1 (*n* = 85)	Q2 (*n* = 85)	Q3 (*n* = 85)	Q4 (*n* = 84)	*p*
Age (years)	68.0 (61.0–75.0)	68.0 (60.0–73.0)	66.0 (61.0–72.0)	65.0 (59.3–71.0)	0.098
Male sex	50 (58.8%)	50 (58.8%)	50 (58.8%)	50 (59.5%)	> 0.999
BMI (kg/m^2^)					**< 0.001**
< 25	84 (98.8%)	76 (89.4%)	68 (80.0%)	53 (63.1%)	
≥ 25	1 (1.2%)	9 (10.6%)	17 (20.0%)	31 (36.9%)	
Hypertension	28 (32.9%)	30 (35.3%)	31 (36.5%)	32 (38.1%)	0.914
Diabetes	18 (21.2%)	18 (21.2%)	27 (31.8%)	21 (25.0%)	0.332
CEA > 5 (ng/ml)	20 (23.5%)	23 (27.1%)	16 (18.8%)	13 (15.5%)	0.267
CA19–9 > 37 (U/L)	54 (63.5%)	48 (56.5%)	49 (57.6%)	60 (71.4%)	0.169
Tumour location					**0.036**
Head	56 (65.9%)	48 (56.5%)	44 (51.8%)	37 (44.0%)	
Body and tail	29 (34.1%)	37 (43.5%)	41 (48.2%)	47 (56.0%)	
Tumour size, cm	3.0 (2.2–3.5)	2.5 (2.0–3.5)	2.5 (2.2–3.8)	2.8 (1.8–3.8)	0.830
Pathological T stage					0.637
T1	11 (12.9%)	16 (18.8%)	16 (18.8%)	14 (16.9%)	
T2	38 (44.7%)	35 (41.2%)	29 (34.1%)	30 (36.1%)	
T3	20 (23.5%)	18 (21.2%)	20 (23.5%)	27 (32.5%)	
T4	16 (18.8%)	16 (18.8%)	20 (23.5%)	12 (14.5%)	
Pathological N stage					**0.044**
N0	44 (51.8%)	43 (50.6%)	34 (40.0%)	54 (64.3%)	
N1	25 (29.4%)	30 (35.3%)	37 (43.5%)	24 (28.6%)	
N2	16 (18.8%)	12 (14.1%)	14 (16.5%)	6 (7.1%)	
Pathological M stage					0.355
M0	81 (95.3%)	78 (91.8%)	77 (90.6%)	81 (96.4%)	
M1	4 (4.7%)	7 (8.2%)	8 (9.4%)	3 (3.6%)	
Tumour differentiations					0.697
Poorly	20 (23.5%)	17 (20.0%)	26 (30.6%)	19 (22.6%)	
Moderately	62 (72.9%)	66 (77.6%)	57 (67.1%)	61 (72.6%)	
Well	3 (3.5%)	2 (2.4%)	2 (2.4%)	4 (4.8%)	
Neoadjuvant therapy	35 (41.2%)	37 (43.5%)	46 (54.1%)	29 (34.5%)	0.077
Adjuvant therapy	66 (77.6%)	76 (89.4%)	82 (96.5%)	80 (95.2%)	**< 0.001**
Neutrophil count (×10^9^/L)	3.9 (2.5–5.3)	3.3 (2.3–4.5)	3.5 (2.5–4.4)	4.0 (2.7–5.0)	0.113
Lymphocyte count (×10^9^/L)	1.0 (0.8–1.3)	1.1 (0.9–1.4)	1.4 (1.0–1.7)	1.6 (1.3–2.0)	**< 0.001**
Haemoglobin (g/L)	111.9 ± 14.8	117.1 ± 16.6	124.5 ± 18.7	133.2 ± 19.1	**< 0.001**
Cholesterol (mmol/L)	4.1 (3.3–4.6)	4.3 (3.7–5.1)	4.2 (3.7–4.9)	4.5 (3.7–5.4)	**0.005**
LDL (mmol/L)	2.1 ± 0.8	2.3 ± 0.7	2.3 ± 0.8	2.5 ± 0.9	**0.004**
HDL (mmol/L)	1.0 (0.8–1.4)	1.1 (0.8–1.4)	1.1 (1.0–1.4)	1.2 (1.0–1.4)	0.078
Albumin (g/L)	36.8 (34.7–40.8)	40.6 (38.3–43.6)	42.9 (40.7–45.2)	44.8 (42.6–46.9)	**< 0.001**
PNI	42.2 (39.3–46.2)	46.6 (43.7–49.8)	50.1 (46.9–52.3)	52.7 (49.8–55.7)	**< 0.001**
SMI (cm^2^/m^2^)	36.2 (32.8–40.9)	41.9 (37.4–45.4)	45.4 (40.1–48.4)	50.6 (45.2–54.2)	**< 0.001**
SATI (cm^2^/m^2^)	34.3 (20.3–52.0)	38.5 (26.5–48.1)	45.9 (33.1–58.4)	43.7 (34.8–57.9)	**< 0.001**
VATI (cm^2^/m^2^)	24.4 (10.1–39.0)	35.1 (21.1–52.5)	40.0 (29.9–56.2)	46.8 (29.9–60.0)	**< 0.001**
SMD (HU)	34.2 (31.1–37.8)	35.0 (30.5–39.8)	35.9 (30.6–40.1)	37.2 (31.8–42.3)	**0.025**

*Note:* Bold indicates that the relevant data has statistical significance.

Abbreviations: BMI, body mass index; CA 19‐9, carbohydrate antigen 19–9; CEA, carcinoembryonic antigen; LDL, low‐density lipoprotein; HDL, high‐density lipoprotein; PNI, prognostic nutritional index; SMI, skeletal muscle index; SATI, subcutaneous adipose tissue index; VATI, visceral adipose tissue index; SMD, skeletal muscle density.

### 3.2 Impact of SMI × PNI on Overall and Disease‐Free Survival in Patients With PDAC

**FIGURE 4:**
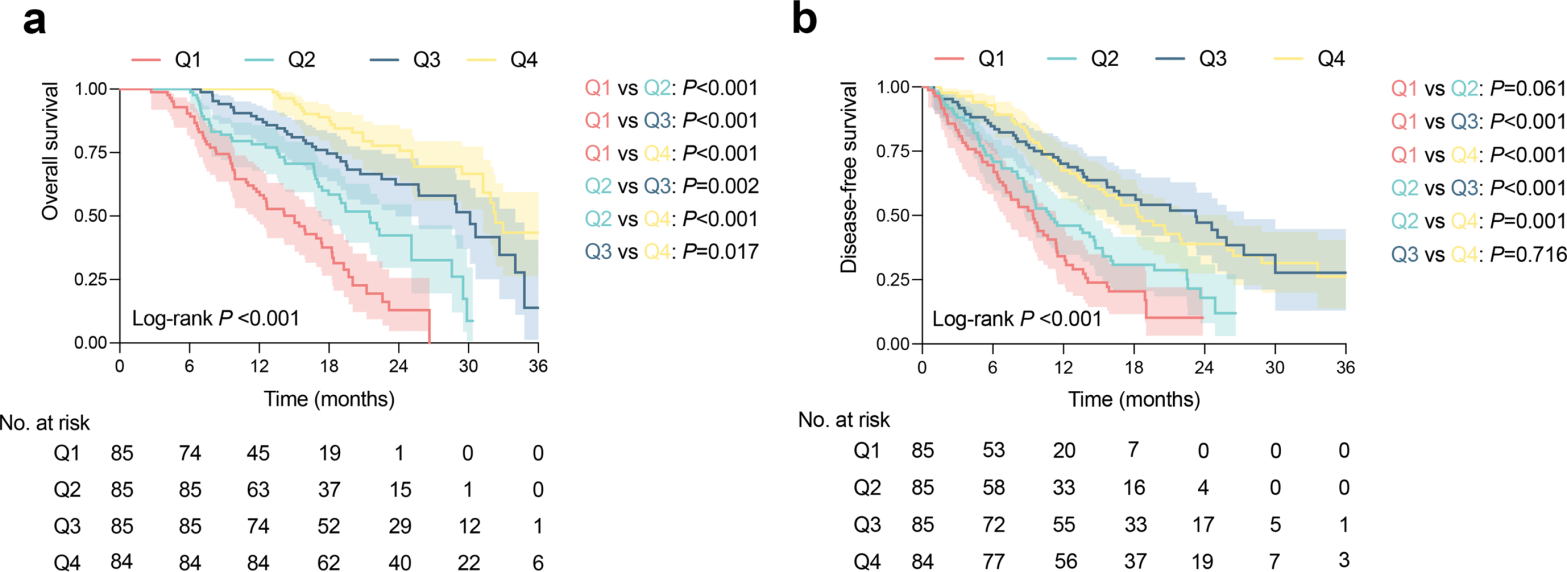
Overall survival (a) and disease‐free survival (b) Kaplan–Meier curves for the patients with PDAC categorized by quartiles of SMI × PNI. SMI, skeletal muscle index; PNI, prognostic nutritional index.

The overall survival curves for the patients categorized by quartiles of SMI × PNI are demonstrated in Figure 4a. Kaplan–Meier analysis revealed significant differences in OS among the SMI × PNI groups (Q1—58.2%, Q2—78.3%, Q3—88.1%, Q4—100%), respectively (*p* overall < 0.001; Q1 vs. Q2 *p* < 0.001; Q1 vs. Q3 *p* < 0.001; Q1 vs. Q4 *p* < 0.001; Q2 vs. Q3 *p* = 0.002; Q2 vs. Q4 *p* < 0.001; Q3 vs. Q4 *p* = 0.017). The median survival time for patients in Q1, Q2, Q3, Q4 groups was 15.0 months, 21.5 months, 30.1 months, and 32.3 months, respectively. Similarly, notable differences in DFS were also observed across the four groups (Figure 4b, *p* overall < 0.001, Q1 vs. Q2 *p* = 0.061; Q1 vs. Q3 *p* < 0.001; Q1 vs. Q4 *p* = 0.001; Q2 vs. Q3 *p* < 0.001; Q2 vs. Q4 *p* = 0.001; Q3 vs. Q4 *p* = 0.716).

Subsequently, the correlation between elevated SMI × PNI levels and OS and DFS in patients with PDAC was further explored using restricted cubic splines (Figure S3a). Based on the adjusted data, a non‐linear association was observed between SMI × PNI levels and OS (*p* overall < 0.001, *p* non‐linear = 0.047). However, no discernible non‐linear relationship was found between elevated SMI × PNI levels and DFS, with non‐linear *p* values of 0.278 (Figure S3b).

### 3.3 Association Between Body Composition Parameters and Prognosis of PDAC

To investigate the prognostic ability of body composition parameters and their combinations with PNI, we divided patients into different subgroups by sex‐specific quartiles of each body composition parameter, including SMI, SATI, VATI and SMD and their combination with PNI. The univariate Cox regression analysis identified age, CEA levels, CA19‐9 levels, tumour location, maximum tumour size, pathological TNM stage, tumour differentiation, receipt of adjuvant therapy, lymphocyte count and haemoglobin levels as significant predictors of OS (Table S3). After adjusting for these factors and potential confounders, the association between these body composition parameters and OS remained highly significant (all *p* overall < 0.05) (Table 2). Notably, using the SMI × PNI levels in Q1 as the reference group, the hazard ratios (HRs) for OS in Q2, Q3 and Q4 were 0.533 (95% CI, 0.349–0.813), 0.205 (95% CI, 0.126–0.336) and 0.130 (95% CI, 0.071–0.235), respectively, demonstrating a robust independent association between SMI × PNI and OS. Similarly, after adjusting for these confounders, SMI × PNI levels was found to be independently correlated with the early tumour recurrence in patients with PDAC (*p* overall < 0.001; Q1 vs. Q2, *p* = 0.075; Q1 vs. Q3, *p* < 0.001; Q1 vs. Q4, *p* < 0.001) (Table 2).

**TABLE 2:** Univariate and multivariate analysis of body composition parameters and prognosis in patients with PDAC.

	Overall survival				Disease free survival			
	Unadjusted		Adjusted		Unadjusted		Adjusted	
Variables	HR (95% CI)	*p* value	HR (95% CI)	*p* value	HR (95% CI)	*p* value	HR (95% CI)	*p* value
**SMI**		< 0.001		< 0.001		0.001		0.011
Q1	Reference		Reference		Reference		Reference	
Q2	0.780 (0.521–1.168)	0.227	0.801 (0.523–1.229)	0.310	0.921 (0.635–1.335)	0.662	0.973 (0.654–1.448)	0.894
Q3	0.512 (0.336–0.782)	0.002	0.537 (0.332–0.868)	0.011	0.617 (0.421–0.903)	0.013	0.626 (0.410–0.957)	0.030
Q4	0.292 (0.183–0.466)	< 0.001	0.316 (0.188–0.532)	< 0.001	0.496 (0.337–0.729)	< 0.001	0.554 (0.360–0.854)	0.007
*p* for trend		< 0.001		< 0.001		< 0.001		0.001
SATI		< 0.001		0.002		0.047		0.077
Q1	Reference		Reference		Reference		Reference	
Q2	0.684 (0.459–1.019)	0.062	0.688 (0.452–1.048)	0.082	0.761 (0.521–1.111)	0.157	0.755 (0.510–1.118)	0.160
Q3	0.347 (0.218–0.553)	< 0.001	0.374 (0.228–0.613)	< 0.001	0.573 (0.388–0.848)	0.005	0.613 (0.408–0.922)	0.019
Q4	0.585 (0.390–0.877)	0.009	0.621 (0.380–1.016)	0.058	0.818 (0.565–1.185)	0.288	0.928 (0.609–1.415)	0.729
*p* for trend		< 0.001		0.005		0.166		0.484
VATI		0.001		0.003		0.573		0.831
Q1	Reference		Reference		Reference		Reference	
Q2	0.637 (0.420–0.965)	0.033	0.830 (0.530–1.301)	0.417	0.792 (0.537–1.169)	0.241	0.998 (0.656–1.517)	0.991
Q3	0.514 (0.340–0.777)	0.002	0.504 (0.316–0.806)	0.004	0.886 (0.609–1.289)	0.528	0.927 (0.609–1.411)	0.724
Q4	0.427 (0.275–0.662)	< 0.001	0.426 (0.256–0.709)	0.001	0.787 (0.538–1.151)	0.217	0.833 (0.537–1.293)	0.415
*p* for trend		< 0.001		< 0.001		0.334		0.365
**SMD**		0.106		0.110		0.331		0.460
Q1	Reference		Reference		Reference		Reference	
Q2	0.936 (0.624–1.405)	0.750	1.159 (0.735–1.828)	0.526	0.853 (0.586–1.242)	0.407	0.894 (0.597–1.339)	0.588
Q3	0.596 (0.382–0.929)	0.022	0.697 (0.422–1.150)	0.157	0.709 (0.485–1.038)	0.077	0.738 (0.482–1.130)	0.162
Q4	0.778 (0.513–1.178)	0.236	1.202 (0.720–2.005)	0.482	0.782 (0.539–1.135)	0.197	0.966 (0.608–1.533)	0.883
*p* for trend		0.079		0.791		0.131		0.633
**SMI** × **PNI**		< 0.001		< 0.001		< 0.001		< 0.001
Q1	Reference		Reference		Reference		Reference	
Q2	0.475 (0.320–0.705)	< 0.001	0.533 (0.349–0.813)	0.004	0.691 (0.481–0.995)	0.047	0.706 (0.481–1.036)	0.075
Q3	0.231 (0.149–0.359)	< 0.001	0.205 (0.126–0.336)	< 0.001	0.331 (0.221–0.494)	< 0.001	0.292 (0.187–0.457)	< 0.001
Q4	0.127 (0.077–0.209)	< 0.001	0.130 (0.071–0.235)	< 0.001	0.358 (0.243–0.529)	< 0.001	0.356 (0.223–0.569)	< 0.001
*p* for trend		< 0.001		< 0.001		< 0.001		< 0.001
**SATI** × **PNI**		< 0.001		< 0.001		0.002		0.016
Q1	Reference		Reference		Reference		Reference	
Q2	0.496 (0.329–0.748)	0.001	0.575 (0.369–0.895)	0.014	0.631 (0.433–0.919)	0.017	0.648 (0.434–0.969)	0.034
Q3	0.363 (0.237–0.556)	< 0.001	0.388 (0.245–0.615)	< 0.001	0.482 (0.328–0.708)	< 0.001	0.529 (0.351–0.798)	0.002
Q4	0.426 (0.282–0.643)	< 0.001	0.437 (0.257–0.745)	0.002	0.680 (0.472–0.982)	0.039	0.779 (0.499–1.216)	0.271
*p* for trend		< 0.001		< 0.001		0.023		0.134
**VATI** × **PNI**		< 0.001		0.001		0.370		0.428
Q1	Reference		Reference		Reference		Reference	
Q2	0.655 (0.435–0.988)	0.044	0.722 (0.466–1.118)	0.145	0.840 (0.573–1.231)	0.371	1.028 (0.683–1.548)	0.893
Q3	0.473 (0.313–0.717)	< 0.001	0.500 (0.314–0.795)	0.003	0.795 (0.544–1.162)	0.236	0.830 (0.542–1.269)	0.389
Q4	0.373 (0.240–0.581)	< 0.001	0.361 (0.213–0.611)	< 0.001	0.714 (0.488–1.045)	0.083	0.724 (0.458–1.144)	0.166
*p* for trend		< 0.001		< 0.001		0.086		0.118
**SMD × PNI**		< 0.001		0.123		0.008		0.330
Q1	Reference		Reference		Reference		Reference	
Q2	0.440 (0.285–0.681)	< 0.001	0.590 (0.368–0.946)	0.028	0.707 (0.480–1.041)	0.079	0.866 (0.564–1.329)	0.511
Q3	0.609 (0.411–0.903)	0.014	0.666 (0.415–1.069)	0.092	0.985 (0.686–1.414)	0.933	1.042 (0.687–1.582)	0.845
Q4	0.411 (0.268–0.630)	< 0.001	0.604 (0.355–1.029)	0.064	0.562 (0.380–0.831)	0.004	0.711 (0.437–1.156)	0.169
*P* for trend		< 0.001		0.072		0.026		0.298

*Note:* Adjusted for: age, sex, neoadjuvant therapy, adjuvant therapy, BMI, pathological TNM stage, haemoglobin, CEA levels, CA19‐9 levels, tumour differentiation, tumour location, maximal tumour size.

Abbreviations: SMI, skeletal muscle index; SATI, subcutaneous adipose tissue index; VATI, visceral adipose tissue index; SMD, skeletal muscle density; PNI, prognostic nutritional index; HR, hazard ratio; CI, confidence interval.

**FIGURE 5:**
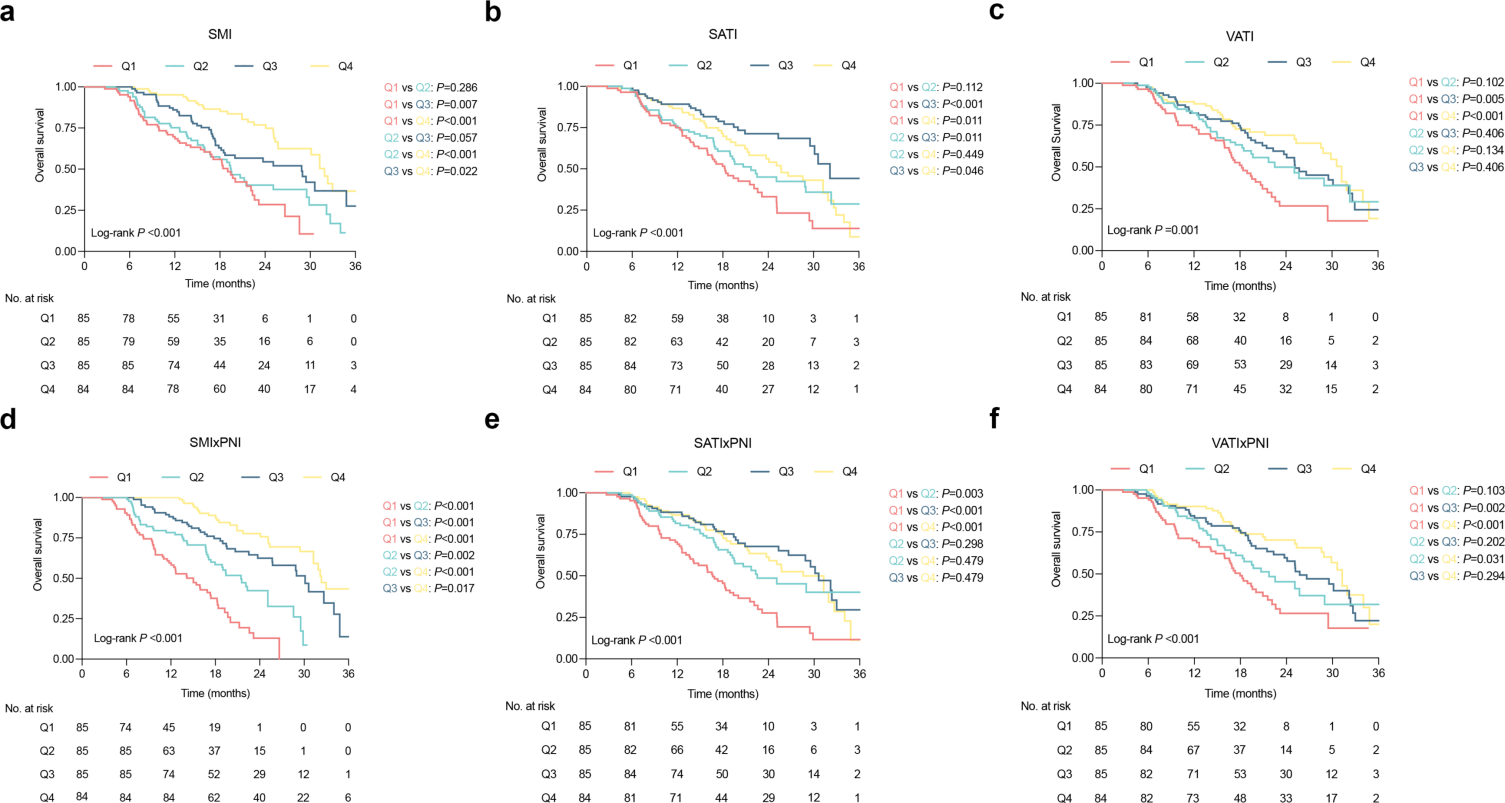
Overall survival Kaplan–Meier curves for patients stratified by sex‐specific quartiles of body composition parameters and the combinations with PNI in the training cohort. Each subfigure was stratified by sex‐specific quartiles of: (a) SMI; (b) SATI; (c) VATI; (d) SMI × PNI; (e) SATI×PNI; (f) VATI×PNI. SMI, skeletal muscle index; SATI, subcutaneous adipose tissue index; VATI, visceral adipose tissue index; PNI, prognostic nutritional index.

Since these indices have been demonstrated as independent risk factors for OS, six models were constructed accordingly. For SMI × PNI levels in the training cohort, each quartile was divided into 1900, 2235 and 2560 in male patients and 1610, 1820 and 2050 in female patients, respectively. The sex‐specific cutoff values for each body composition parameter and their combinations with PNI in the training cohort are detailed in Table S4. The overall survival curves for patients stratified by sex‐specific quartiles of different indices are illustrated in Figure 5 and Figure S4. The results reveal that the SMI × PNI model exhibited superior performance for OS compared to other models (c‐statistics: 0.767) (Table 3). In addition, the SMI × PNI model demonstrated a lower AICc value of 1648.8, suggesting better predictive ability for patient survival (Table 3).

**TABLE 3:** The predictive and discriminatory power of different body composition parameters and the combinations with PNI.

	Training cohort			Validation cohort		
	AICc	c‐Statistics	*p* ^a^	AICc	c‐Statistics	*p* ^a^
SMI	1697.5	0.677	2.98 × 10^−6^	478.3	0.678	1.06 × 10^−3^
SATI	1718.5	0.612	2.68 × 10^−6^	481.5	0.643	1.05 × 10^−2^
VATI	1714.0	0.620	5.92 × 10^−6^	487.1	0.583	5.39 × 10^−4^
SMD	1726.4	0.567	2.10 × 10^−8^	486.1	0.584	1.17 × 10^−3^
SMI × PNI	1648.8	0.767	Reference	457.6	0.770	Reference
SATI×PNI	1710.5	0.642	3.70 × 10^−5^	474.9	0.695	6.28 × 10^−2^
VATI×PNI	1707.9	0.649	7.62 × 10^−5^	484.4	0.620	1.27 × 10^−3^
SMD × PNI	1717.5	0.629	1.62 × 10^−6^	470.8	0.717	1.08 × 10^−2^

Abbreviations: SMI, skeletal muscle index; SATI, subcutaneous adipose tissue index; VATI, visceral adipose tissue index; SMD, skeletal muscle density; PNI, prognostic nutritional index.

^a^ The comparison of c‐statistics was performed using a Z‐test with Noether's variance estimation.

### 3.4 Stratified Analysis of SMI × PNI in Patients with PDAC

Stratified analysis was conducted to further explore the correlations between the prognosis of patients with PDAC and SMI × PNI levels. The results, stratified by age, sex, BMI, tumour location, tumour stage, tumour differentiation, CA 19‐9 levels and neoadjuvant therapy, are displayed in Figure S5. Strikingly, elevated preoperative SMI × PNI levels were associated with favourable clinical outcomes across all stratified analyses.

### 3.5 External Validation of SMI × PNI as a Prognostic Tool

In addition, 124 patients (76 males and 48 females) were enrolled retrospectively at the First Affiliated Hospital of Wenzhou Medical University, forming an external validation cohort. The overall survival curves for this cohort, stratified by sex‐specific quartiles of different indices from the training cohort, are illustrated in Figure S4 and Figure S6. As anticipated, the SMI × PNI model demonstrated superior predictive power for OS compared to other models (c‐statistics: 0.770; AICc: 457.6) (Table 3). Thus, SMI × PNI may serve as a more effective prognostic indicator for OS with patients with PDAC compared to other indices.

## 4 Discussion

In light of our findings, we systematically and comprehensively examined the relationship between body composition parameters, their integration with nutritional assessment and clinical outcomes in 576 patients from two specialized centres. Our results demonstrated that elevated levels of SMI × PNI were significantly associated with prolonged OS and DFS among patients with PDAC who underwent radical resection, and SMI × PNI showed superior predictive capability for OS compared with other body composition parameters such as SATI, VATI, SMD and their combinations with PNI. Additionally, this correlation remained robust even after stratification by age, sex and other relevant factors. The predictive value of SMI × PNI was also validated in an independent external cohort, further supporting its utility as a reliable prognostic marker.

Given the widespread use of CT in patients with PDAC, the assessment of body composition parameters obtained from a cross‐sectional CT image at the L3 level has been extensively investigated [23]. Ninomiya et al. reported that, in a cohort of 265 patients from East Asia who underwent curative surgery for PDAC, sarcopenia was identified as an independent prognostic factor in those with a BMI greater than 22 [24]. Besides, in patients with advanced pancreatic cancer receiving palliative therapy or best supportive care, a rapid decline in VAT over one month was closely associated with poorer survival outcomes [25].

However, the heterogeneous results regarding the association between body composition parameters were observed in different retrospective studies. For instance, Paiella et al. analysed 121 patients who underwent neoadjuvant therapy followed by pancreatectomy and found that a lower pre‐therapy SMI (*p* = 0.035) and an increase in SAT during therapy (*p* = 0.043) were significantly associated with a higher risk of major postoperative complications, whereas VAT was not [26]. Besides, Min Woo Lee and colleagues reported that in patients with metastatic pancreatic cancer, body composition primarily changed during the first 2 months after the initiation of chemotherapy, and the prognostic factors associated with OS differed between males and females [27]. In a single‐centre study involving 101 patients with advanced gastric cancer treated with immunotherapy, low SMI was identified as an independent risk factor for earlier tumour regression, while patients with high SATI were more likely to experience adverse events [28]. In our study, patients with PDAC with lower SMI, SATI and VATI levels exhibited shorter OS rates. However, only low SMI and SATI were linked to earlier tumour recurrence, while low VATI showed no such association. The findings suggest that the impact of body composition parameters on PDAC prognosis may vary depending on the specific parameter analysed, highlighting the need for further validation in larger, more diverse populations.

PNI, composed of albumin and lymphocyte counts, is a well‐established marker for nutritional status and cancer prognosis. A meta‐analysis of 14 studies indicated that preoperative PNI level in patients with PDAC serves as a significant prognostic factor [29]. In addition, Shimizu et al. revealed that patients with unresectable pancreatic cancer with pre‐treatment PNI < 43 were more likely to suffer unfavourable clinical outcomes [30]. Albumin functions as a vital nutrient reservoir, playing crucial roles in physiological processes such as immune regulation and fluid balance [31]. Tumour progression is frequently associated with hypoproteinemia, driven by factors such as reduced hepatic synthesis, inadequate nutritional intake and metabolic disturbances in patients with cancer [32]. Lymphocytes are widely recognized as critical markers for tumour‐related nutritional status and essential components of both the immune system and the tumour microenvironment [33]. While immune surveillance is crucial for anti‐tumour immunity, tumour cells can secrete factors that suppress CD4^+^ and CD8^+^ lymphocyte activity, resulting in lymphocytopenia and facilitating immune evasion [34]. Therefore, enhancing lymphocyte count and function through nutritional support may strengthen immune surveillance against tumours, potentially improving prognosis in PDAC.

Integrating body composition parameters with other indices, such as nutritional or inflammation‐related markers, offers both advantages and challenges in disease prediction. On the one hand, this synergy can enhance the predictive power of disease models. For example, Liao et al. demonstrated that combining sarcopenic obesity with the lymphocyte‐to‐monocyte ratio (LMR) index yielded excellent predictive performance for patients with hepatocellular carcinoma undergoing liver resection, outperforming several traditional models [35]. Additionally, such integrative approaches facilitate disease stratification. As observed by Yerim Kim et al., in patients with non‐metastatic colorectal cancer, those with high albumin and high SMD levels exhibited significantly better 5‐year survival rates compared to individuals with low albumin and low SMD levels [4]. On the other hand, however, the combined prediction approach has its limitations. Blood‐based biomarkers, while clinically accessible, are subject to transient fluctuations influenced by physiological states, comorbidities or therapeutic interventions. The neutrophil‐to‐lymphocyte ratio (NLR), for example, demonstrates dynamic variability affected by acute stress, pharmacological agents and age‐related immune remodelling, potentially compromising its reliability as a standalone prognostic indicator [36]. These limitations underscore the necessity for rigorous temporal standardization of biomarker measurements and cautious interpretation within clinical contexts.

In the context of cancer cachexia, the interplay between muscle mass, nutritional status and tumour‐induced chronic inflammation is critical. Tumour microenvironment‐driven cytokines, such as IL‐6 and TNF‐α, orchestrate systemic catabolic processes that link muscle wasting to nutritional dysregulation. Recent studies have demonstrated that IL‐6 not only initiates the wasting of muscle and adipose tissue but also inhibits hepatic albumin production [37]. TNF‐α induces chronic systemic inflammation, leading to fat redistribution to the intra‐abdominal area and fatty infiltration of skeletal muscles, ultimately diminishing overall strength and functionality [38]. Additionally, TNF‐α selectively inhibits the gene expression of albumin, playing a critical role in the development of malnutrition in pancreatic cancer [39]. This multi‐target disruption suggests that isolated body composition metrics may inadequately capture systemic pathological burdens, whereas combining nutritional indices could reflect both protein–energy imbalance and inflammatory dysregulation, therefore the combination of SMI and PNI may exert a synergistic effect on prognostic stratification. In our study, while SMI, SATI, VATI, SMD and their integrations with PNI were significant prognostic factors in multivariate analysis, the discriminatory and predictive ability of SMI × PNI outperformed all other variables.

Several limitations of our study should be noted. Firstly, its retrospective nature does not allow for avoiding selection bias, as patients with complete preoperative assessments and those not lost to follow‐up in the short term were more likely to be included. Future studies may mitigate these limitations by employing a prospective design with pre‐specified inclusion criteria, recruiting larger and more diverse patient populations across multiple centres. Secondly, as each patient received different pre‐ and post‐operative treatments based on their pathological stage, we could not assess the specific impact of these treatments on individual prognosis. Instead, we performed multivariate analysis adjusting for these variables, and the results remained consistent. At last, our study only examined body composition at a single time point, and did not account for time‐varying changes in a longitudinal analysis. Further prospective studies that reflect muscle strength, physical performance and long‐term changes are required.

In conclusion, our study found that the combination of SMI and PNI represents a novel and valuable prognostic indicator of overall survival in patients with resected PDAC, demonstrating superior prognostic value compared to SMI, SATI, VATI or SMD alone. If both SMI and PNI are available, SMI × PNI may be a useful tool for predicting clinical outcomes, optimizing patient management, and improving survival.

## Ethics Statement

The studies involving human participants were reviewed and approved by the Ethical Decision Committee of the Research Administration at the First Affiliated Hospital, Zhejiang University School of Medicine and the First Affiliated Hospital of Wenzhou Medical University. This study was conducted in accordance with the Helsinki Declaration.

## Conflicts of Interest

The authors declare no conflicts of interest.

## Supporting information


**Figure S1** Comparison of body composition parameters and the combinations with PNI according to sex.
**Figure S2.** Comparison of SMD and the combinations with PNI according to sex.
**Figure S3.** Nonlinear association of SMI × PNI levels with overall survival (a) and disease‐free survival (b) in patients with PDAC.
**Figure S4.** Overall survival Kaplan–Meier curves for patients stratified by SMD and the combinations with PNI in the training and external validation cohort.
**Figure S5.** Overall survival of patients with pancreatic cancer based on quartiles of SMI × PNI stratified by age, gender, BMI, tumour location, neoadjuvant therapy, tumour stage, tumour differentiation and CA19–9 levels.
**Figure S6.** Overall survival Kaplan–Meier curves for patients stratified by sex‐specific quartiles of body composition parameters and the combinations with PNI in the external validation cohort.
**Table S1.** Baseline characteristics of patients with PDAC in our study.
**Table S2.** Baseline characteristics of patients with PDAC in the training cohort.
**Table S3.** Univariate analysis of baseline characteristics and overall survival in patients with PDAC.
**Table S4.** The sex‐specific cutoff values for each body composition parameter and their combinations with PNI in the training cohort.
