# Supplementary material for: Integrating Body Composition and Nutritional Indices: A Novel Prognostic Tool for Survival in Pancreatic Cancer
Source: J Cachexia Sarcopenia Muscle. 2025 Jul 9;16(4):e70006. doi: 10.1002/jcsm.70006 (PMC12238902; doi:10.1002/jcsm.70006)
Supplement: Supplementary file 1 — Figure S1 Comparison of body composition parameters and the combinations with PNI according to sex. Figure S2. Comparison of SMD and the combinations with PNI according to sex. Figure S3. Nonlinear association of SMI × PNI levels with overall survival (a) and disease‐free survival (b) in patients with PDAC. Figure S4. Overall survival Kaplan–Meier curves for patients stratified by SMD and the combinations with PNI in the training and external validation cohort. Figure S5. Overall survival of patients with pancreatic cancer based on quartiles of SMI × PNI stratified by age, gender, BMI, tumour location, neoadjuvant therapy, tumour stage, tumour differentiation and CA19–9 levels. Figure S6. Overall survival Kaplan–Meier curves for patients stratified by sex‐specific quartiles of body composition parameters and the combinations with PNI in the external validation cohort. Table S1. Baseline characteristics of patients with PDAC in our study. Table S2. Baseline characteristics of patients with PDAC in the training cohort. Table S3. Univariate analysis of baseline characteristics and overall survival in patients with PDAC. Table S4. The sex‐specific cutoff values for each body composition parameter and their combinations with PNI in the training cohort. [file JCSM-16-e70006-s001.docx]

**Supplementary Materials**

**Integrating Body Composition and Nutritional Indices: A Novel Prognostic Tool for Survival in Pancreatic Cancer**

Yiting Xu^1,2,#^, Yang Chen^1,2,#^, Gaowei Jin^1,2,#^, Chenrui Yao^3^, Yangyang, Wang^1,2^, Ziyang Wei^1,2^, Zhihang Cai^1,2^, Xuanhao Gu^1,2^, Binbin Deng^4^, Peilu Wang^5^, Yuxiong Feng^6,7^, Qi Zhang^1,2,8,9,10*^, Tingbo Liang^1,2,8,9,10*^

1. Department of Hepatobiliary and Pancreatic Surgery, The First Affiliated Hospital, Zhejiang University School of Medicine, Hangzhou, China
2. Zhejiang Provincial Key Laboratory of Pancreatic Disease, The First Affiliated Hospital, Zhejiang University School of Medicine, Hangzhou, China
3. Department of Gastroenterology, The First Affiliated Hospital of Wenzhou Medical University, Wenzhou, China
4. Department of Rehabilitation Medicine, The First Affiliated Hospital of Wenzhou Medical University, Wenzhou, Zhejiang, China
5. Department of Nutrition and Food Hygiene, School of Public Health, Institute of Nutrition, Fudan University, Shanghai, China
6. Zhejiang Key Laboratory of Pancreatic Disease, The First Affiliated Hospital, Zhejiang Key Laboratory of Frontier Medical Research on Cancer Metabolism, Institute of Translational Medicine, Zhejiang University School of Medicine, Hangzhou, China
7. Institute of Fundamental and Transdisciplinary Research, Cancer Center, Zhejiang University, Hangzhou, China
8. The Innovation Center for the Study of Pancreatic Diseases of Zhejiang Province, Hangzhou, China
9. MOE Joint International Research Laboratory of Pancreatic Diseases, The First Affiliated Hospital, Zhejiang University School of Medicine, Hangzhou, China
10. Zhejiang University Cancer Center, Hangzhou, China

***** Corresponding author. Department of Hepatobiliary and Pancreatic Surgery, the First Affiliated Hospital, Zhejiang University School of Medicine, No. 79 Qingchun Road, Hangzhou, 310003, China.

E-mail addresses: qi.zhang@zju.edu.cn (Q. Zhang), liangtingbo@zju.edu.cn (T. Liang).

**#** These authors contributed equally to this work.

Contents

Supplementary Figure 1. Comparison of body composition parameters and the combinations with PNI according to sex.………………....……………....…………...……………...……………...……………3

Supplementary Figure 2. Comparison of SMD and the combinations with PNI according to sex……….4

Supplementary Figure 3. Nonlinear association of SMI×PNI levels with overall survival (a) and disease-free survival (b) in patients with PDAC.……...…………………………...……………….……….……5

Supplementary Figure 4. Overall survival Kaplan-Meier curves for patients stratified by SMD and the combinations with PNI in the training and external validation cohort...………….…………………….6

Supplementary Figure 5. Overall survival of patients with pancreatic cancer based on quartiles of SMI×PNI stratified by age, gender, BMI, tumor location, neoadjuvant therapy, tumor stage, tumor differentiation and CA19-9 levels. ………………………………………………………………………7

Supplementary Figure 6. Overall survival Kaplan-Meier curves for patients stratified by sex-specific quartiles of body composition parameters and the combinations with PNI in the external validation cohort…………………………………………………………………………………………. ….…….8

Supplementary Table 1. Baseline characteristics of PDAC patients in our study….….…………………9

Supplementary Table 2. Baseline characteristics of PDAC patients in the training cohort………………11

Supplementary Table 3. Univariate analysis of baseline characteristics and overall survival in PDAC patients.…………………………………………………………………………………………………13

Supplementary Table 4. The sex-specific cutoff values for each body composition parameter and their combinations with PNI in the training cohort.……………………………………………………….…15

Additional References...…………………………………………………………………………..……16


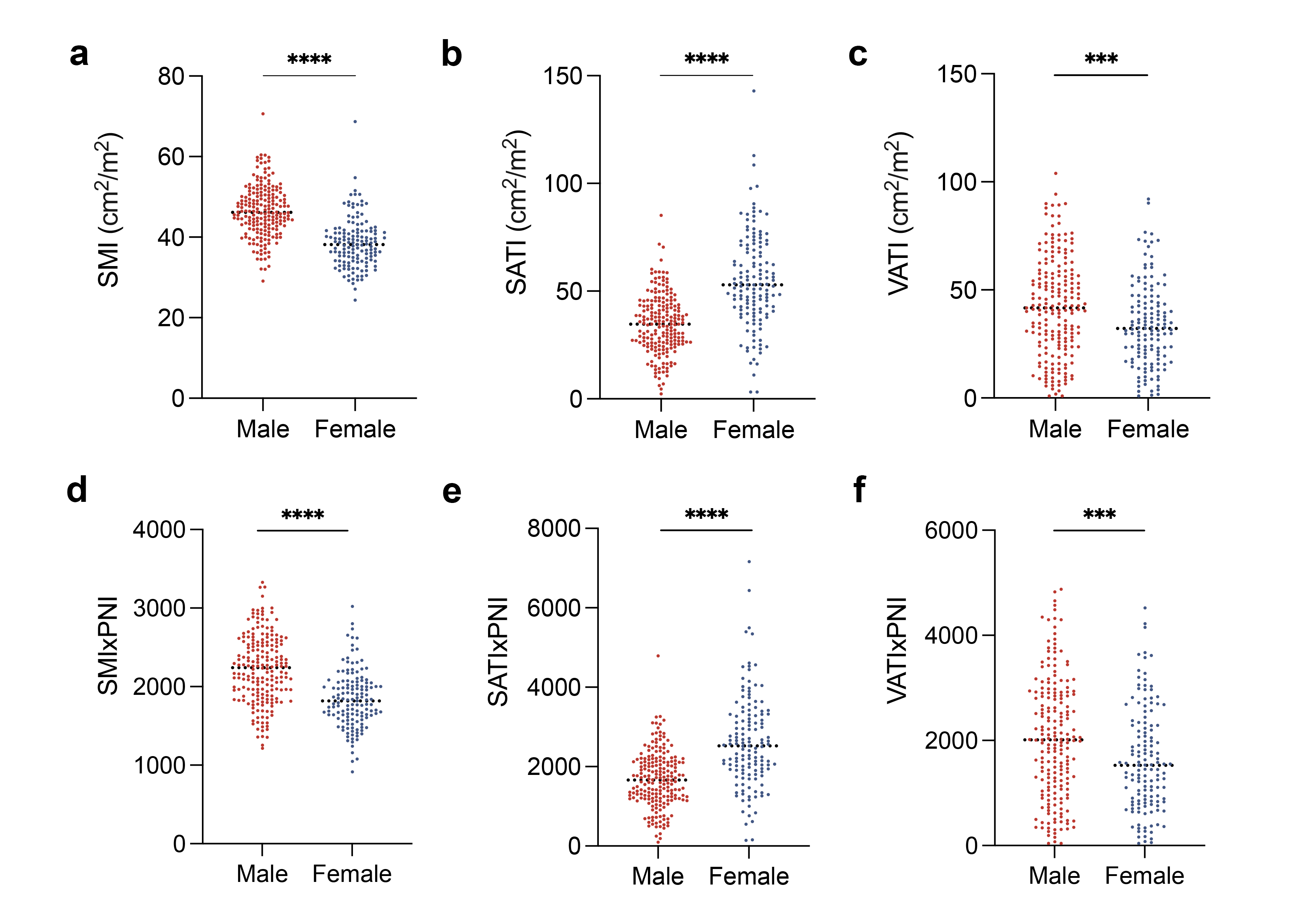


Supplementary Figure 1. Comparison of body composition parameters and the combinations with PNI according to sex. (a) Comparison of SMI by sex; (b) Comparison of SATI by sex; (c) Comparison of VATI by sex; (d) Comparison of SMI×PNI by sex; (e) Comparison of SATI×PNI by sex; (f) Comparison of VATI×PNI by sex. Due to the abnormal distribution of the data, the data was compared using Wilcoxon rank sum test between two groups. ***, *P* <0.001; ****, *P* <0.0001. SMI, skeletal muscle index; SATI, subcutaneous adipose tissue index; VATI, visceral adipose tissue index; PNI, prognostic nutritional index.


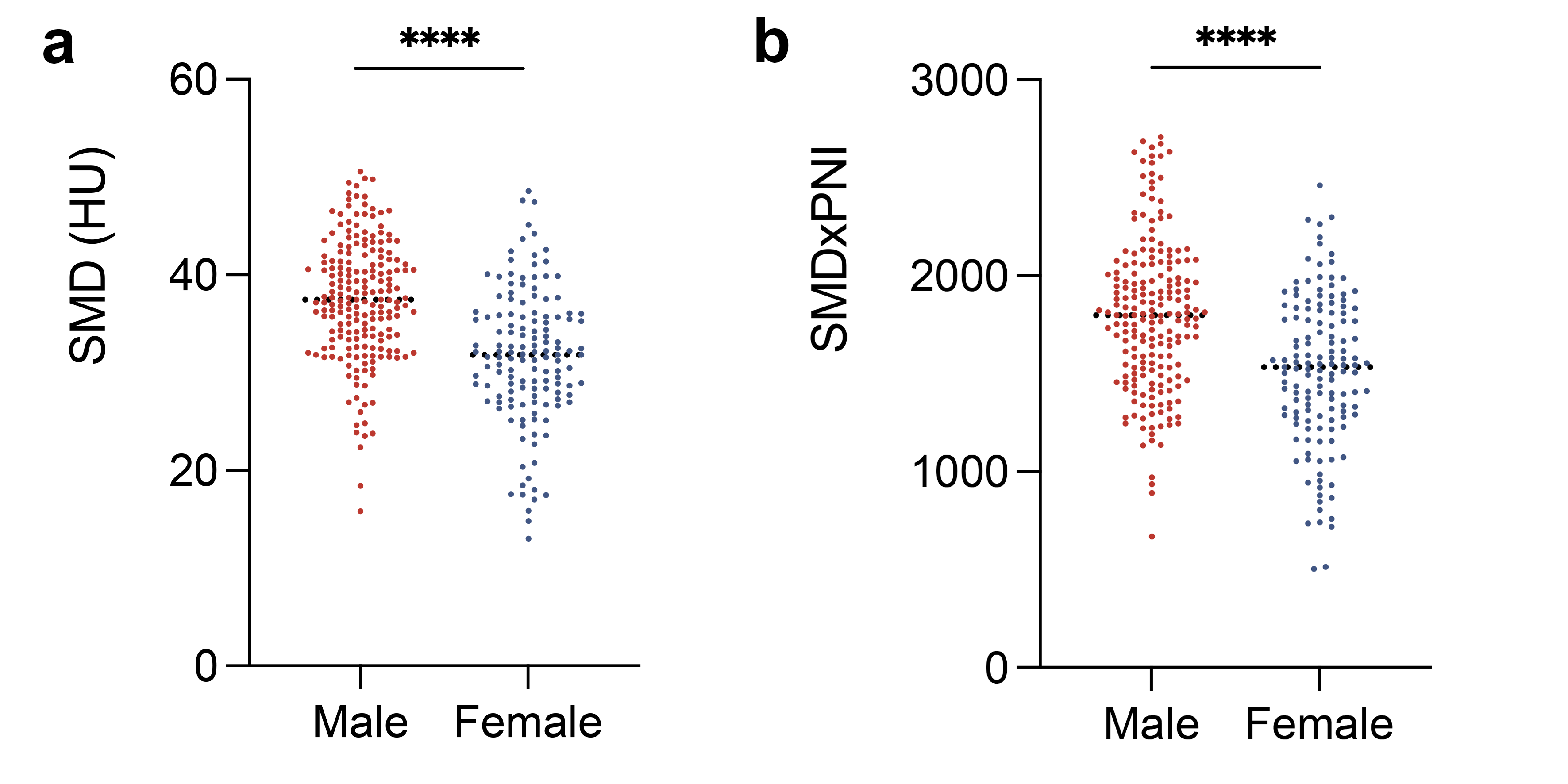


Supplementary Figure 2. Comparison of SMD and the combinations with PNI according to sex. (a) Comparison of SMD by sex; (b) Comparison of SMD×PNI by sex. Due to the abnormal distribution of the data, the data was compared using Wilcoxon rank sum test between two groups. ****, *P* <0.0001. SMD, skeletal muscle density; PNI, prognostic nutritional index.


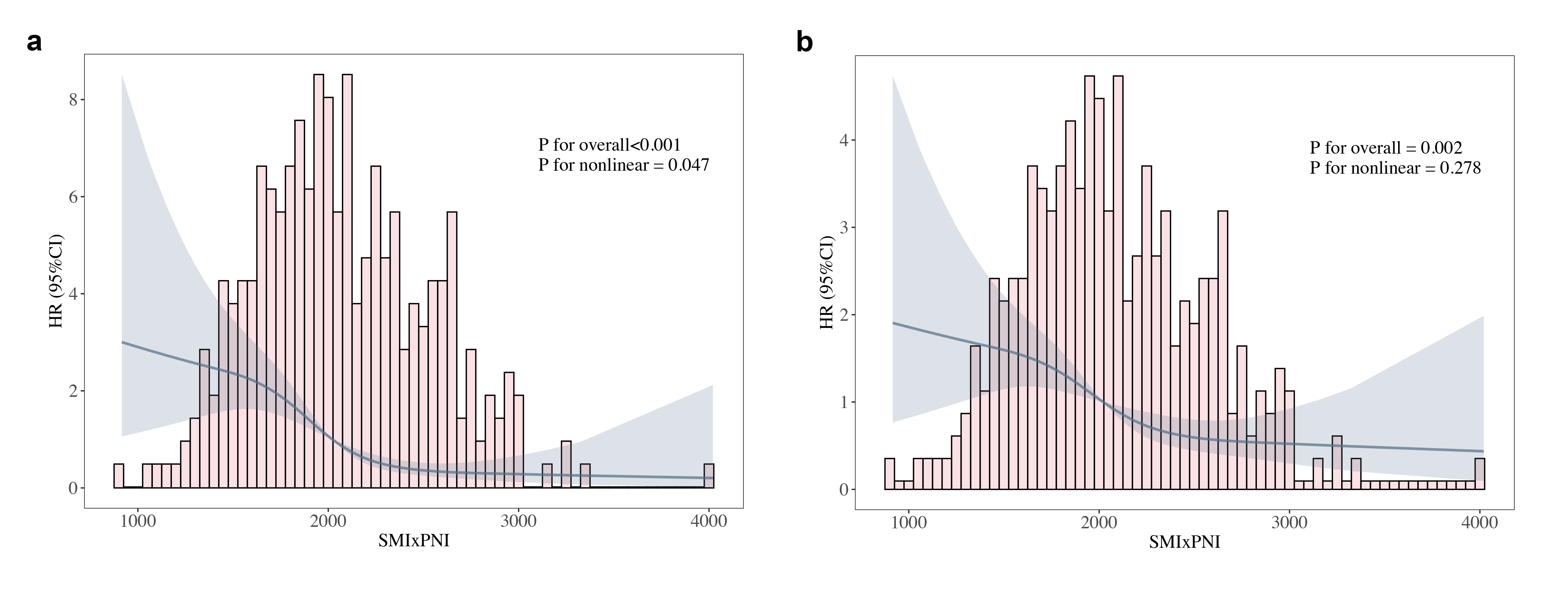


Supplementary Figure 3. Nonlinear association of SMI×PNI levels with overall survival (a) and disease-free survival (b) in patients with PDAC. Adjusted for: age, sex, neoadjuvant therapy, adjuvant therapy, BMI, pathological TNM stage, hemoglobin, CEA levels, CA19-9 levels, tumor differentiation, tumor location, maximal tumor size. SMI, skeletal muscle index; PNI, prognostic nutritional index.


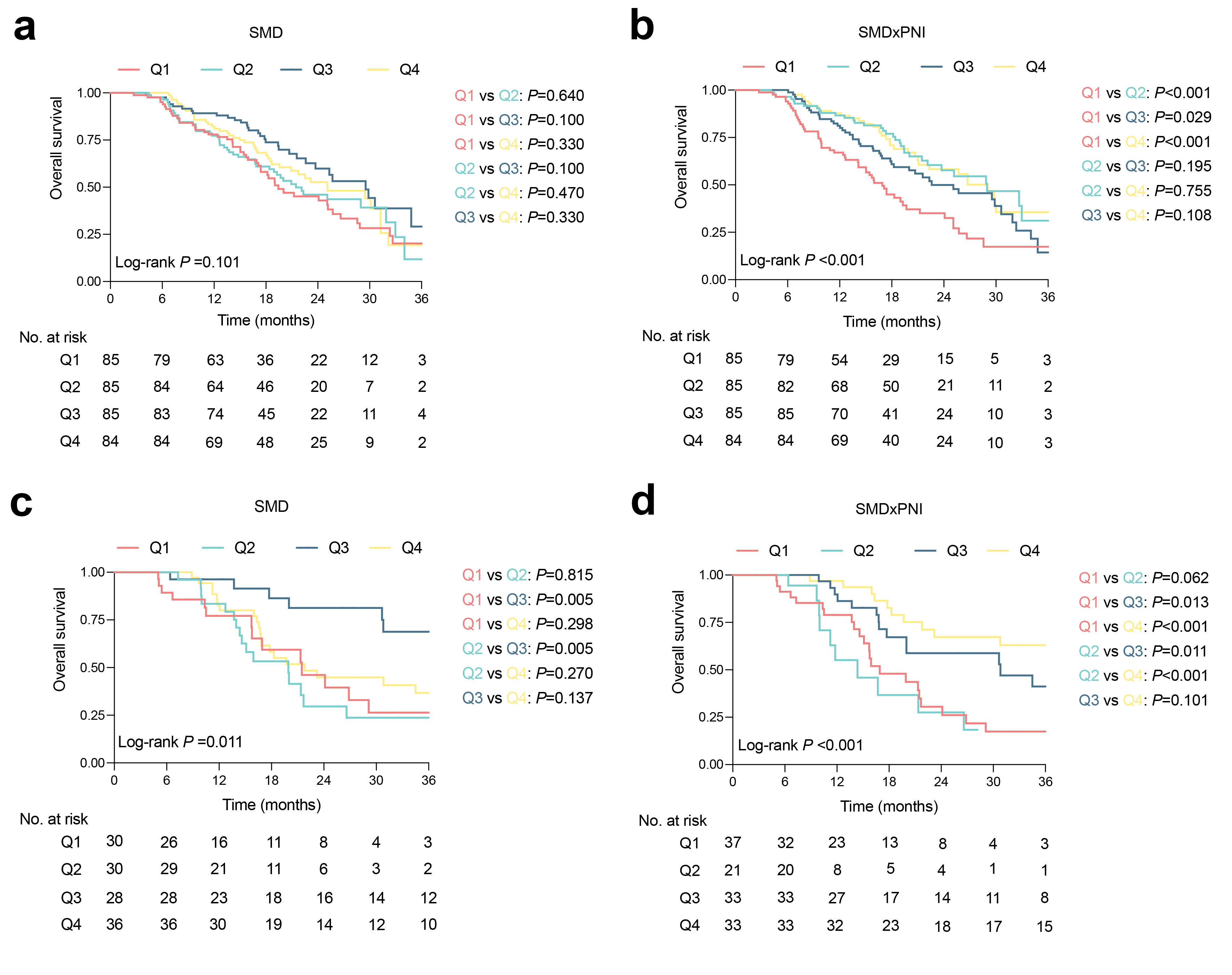


Supplementary Figure 4. Overall survival Kaplan-Meier curves for patients stratified by SMD and the combinations with PNI in the training and external validation cohort. (a) SMD in the training cohort; (b) SMD×PNI in the training cohort; (c) SMD in the external validation cohort; (d) SMD×PNI in the external validation cohort. SMD, skeletal muscle density; PNI, prognostic nutritional index.


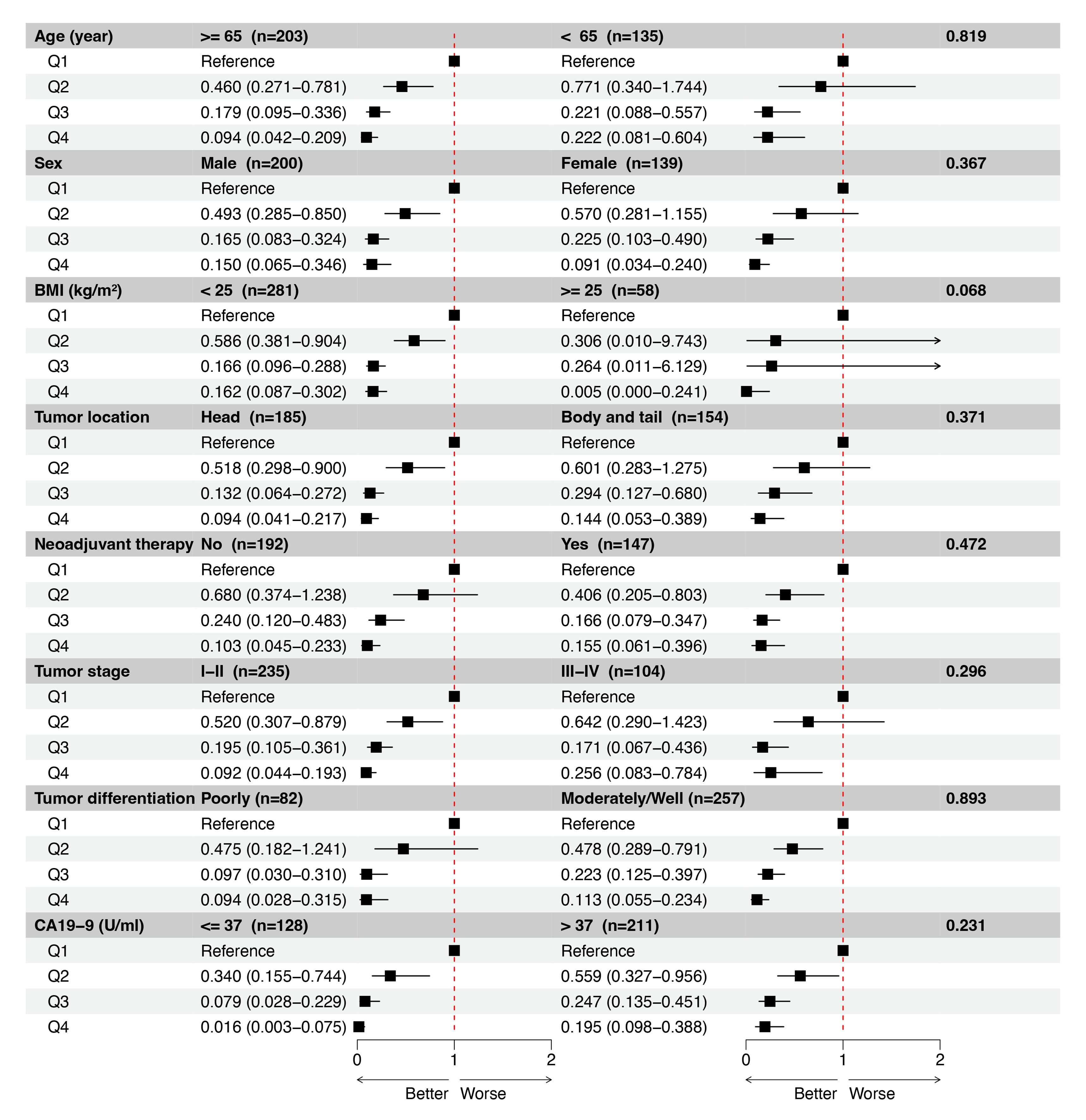


Supplementary Figure 5. Overall survival of patients with pancreatic cancer based on quartiles of SMI×PNI stratified by age, gender, BMI, tumor location, neoadjuvant therapy, tumor stage, tumor differentiation and CA19-9 levels. The analysis is adjusted for: age, sex, neoadjuvant therapy, adjuvant therapy, BMI, pathological TNM stage, hemoglobin, CEA levels, CA19-9 levels, tumor differentiation, tumor location, maximal tumor size. In each case, the analysis is not adjusted for the stratification variable.


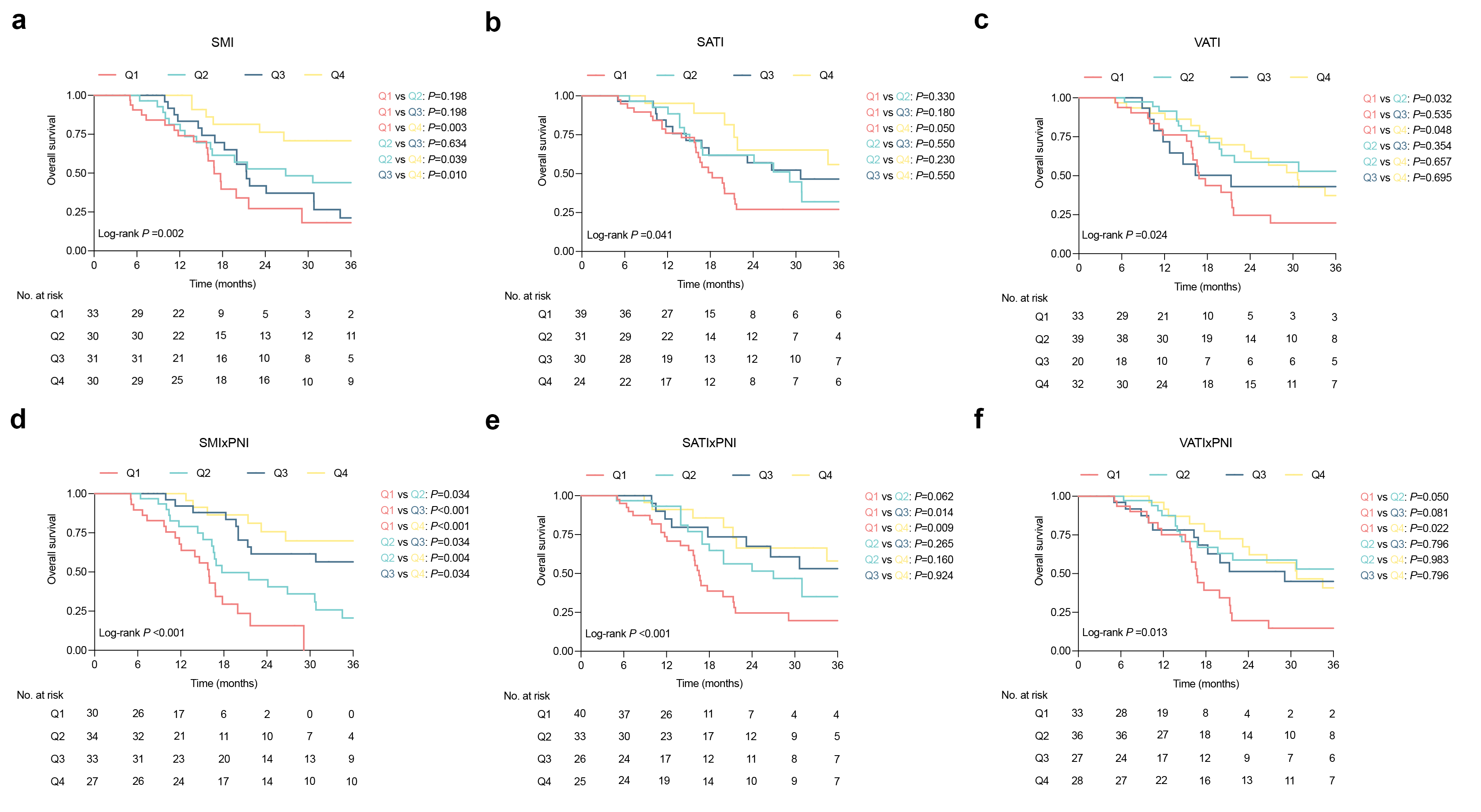


Supplementary Figure 6. Overall survival Kaplan-Meier curves for patients stratified by sex-specific quartiles of body composition parameters and the combinations with PNI in the external validation cohort. Each subfigure was stratified by sex-specific quartiles of: (a) SMI; (b) SATI; (c) VATI; (d) SMI×PNI; (e) SATI×PNI; (f) VATI×PNI. SMI, skeletal muscle index; SATI, subcutaneous adipose tissue index; VATI, visceral adipose tissue index; PNI, prognostic nutritional index.

Supplementary Table 1. Baseline characteristics of PDAC patients in our study.

|  | Training cohort (n=339) | Validation cohort (n=124) | *P* value |
| --- | --- | --- | --- |
| Age (years) | 67.0 (60.0-72.0) | 63.0 (55.3-70.0) | **<0.001** |
| Male sex | 200 (59.0%) | 76 (61.3%) | 0.656 |
| BMI (kg/m^2^) |  |  | 0.379 |
| < 25 | 281 (82.9%) | 107 (86.3%) |  |
| ≥ 25 | 58 (17.1%) | 17 (13.7%) |  |
| Hypertension | 121 (35.7%) | 48 (38.7%) | 0.649 |
| Diabetes | 84 (24.8%) | 44 (35.5%) | 0.079 |
| CEA >5 (ng/ml) | 72 (21.2%) | 40 (32.3%) | 0.058 |
| CA19-9 >37 (U/L) | 211 (62.2%) | 88 (71.0%) | 0.189 |
| Tumor location |  |  | 0.092 |
| Head | 185 (54.6%) | 82 (66.1%) |  |
| Body and tail | 154 (45.4%) | 42 (33.9%) |  |
| Tumor size, cm | 2.8 (2.0-3.6) | 3 (2.5-4.0) | 0.082 |
| Pathological T stage |  |  | **<0.001** |
| T1 | 57 (16.9%) | 12 (9.7%) |  |
| T2 | 132 (39.1%) | 92 (74.2%) |  |
| T3 | 85 (25.1%) | 20 (16.1%) |  |
| T4 | 64 (18.9%) | 0 (0.0%) |  |
| Pathological N stage |  |  | 0.061 |
| N0 | 175 (51.6%) | 84 (67.7%) |  |
| N1 | 116 (34.2%) | 30 (24.2%) |  |
| N2 | 48 (14.2%) | 10 (8.1%) |  |
| Pathological M stage |  |  | 0.621 |
| M0 | 317 (93.5%) | 118 (95.2%) |  |
| M1 | 22 (6.5%) | 6 (4.8%) |  |
| Tumor differentiations |  |  | 0.774 |
| Poorly | 82 (24.2%) | 32 (25.8%) |  |
| Moderately | 246 (72.6%) | 86 (69.4%) |  |
| Well | 11 (3.2%) | 6 (4.8%) |  |
| Neoadjuvant therapy | 147 (43.4%) | 2 (1.6%) | **<0.001** |
| Adjuvant therapy | 304 (89.7%) | 106 (85.5%) | 0.332 |
| Neutrophil count (x10^9^/L) | 3.6 (2.5-4.8) | 3.5 (2.7-5.1) | 0.400 |
| Lymphocyte count (x10^9^/L) | 1.3 (0.9-1.7) | 1.4 (1.1-1.8) | **0.001** |
| Hemoglobin (g/L) | 121.6 ± 19.1 | 124.3 ± 18.5 | 0.175 |
| Cholesterol (mmol/L) | 4.3 (3.6-5.0) | 4.9 (4.1-5.7) | **<0.001** |
| LDL (mmol/L) | 2.3 ± 0.8 | 3.1 ± 1.2 | **<0.001** |
| HDL (mmol/L) | 1.1 (0.9-1.4) | 1.0 (0.6-1.4) | **0.017** |
| Albumin (g/L) | 41.7 (38.2-44.7) | 39.8 (37.1-42.7) | **0.001** |
| PNI | 48.0 (44.1-51.9) | 46.8 (44.1-51.7) | 0.288 |
| SMI (cm^2^/m^2^) | 43.1 (38.0-48.3) | 42.3 (37.7-49.2) | 0.937 |
| SATI (cm^2^/m^2^) | 40.8 (28.3-53.2) | 34.9 (26.4-52.9) | 0.125 |
| VATI (cm^2^/m^2^) | 37.0 (22.6-53.1) | 34.4 (22.2-51.8) | 0.532 |
| SMD (HU) | 35.6 (31.1-40.1) | 35.8 (32.1-39.4) | 0.477 |

Bold indicates that the relevant data has statistical signiﬁcance.

Abbreviations: BMI, body mass index; CA 19-9, carbohydrate antigen 19-9; CEA, carcinoembryonic antigen; LDL, low-density lipoprotein; HDL, high-density lipoprotein; PNI, prognostic nutritional index; SMI, skeletal muscle index; SATI, subcutaneous adipose tissue index; VATI, visceral adipose tissue index; SMD, skeletal muscle density.

Supplementary Table 2. Baseline characteristics of PDAC patients in the training cohort.

|  | Overall (n=339) | Male (n=200) | Female (n=139) | *P* value |
| --- | --- | --- | --- | --- |
| Age (years) | 67.0 (60.0-72.0) | 66.0 (60.0-73.0) | 68.0 (60.0-72.0) | 0.911 |
| BMI (kg/m^2^) |  |  |  | **0.047** |
| < 25 | 281 (82.9%) | 159 (79.5%) | 122 (87.8%) |  |
| ≥ 25 | 58 (17.1%) | 41 (20.5%) | 17 (12.2%) |  |
| Hypertension | 121 (35.7%) | 76 (38.0%) | 45 (32.4%) | 0.288 |
| Diabetes | 84 (24.8%) | 62 (31.0%) | 22 (15.8%) | **0.001** |
| CEA >5 (ng/ml) | 72 (21.2%) | 49 (24.5%) | 23 (16.5%) | 0.078 |
| CA19-9 >37 (U/L) | 211 (62.2%) | 140 (70.0%) | 71 (51.1%) | **<0.001** |
| Tumor location |  |  |  | 0.800 |
| Head | 185 (54.6%） | 108 (54.0%) | 77 (55.4%) |  |
| Body and tail | 154 (45.4%) | 92 (46.0%) | 62 (44.6%) |  |
| Tumor size, cm | 2.8 (2.0-3.6) | 2.8 (2.0-3.8) | 2.5 (2.0-3.5) | 0.129 |
| Pathological T stage |  |  |  | 0.552 |
| T1 | 57 (16.9%) | 33 (16.5%) | 24 (17.4%) |  |
| T2 | 132 (39.1%) | 73 (36.5%) | 59 (42.8%) |  |
| T3 | 85 (25.1%) | 52 (26.0%) | 33 (23.9%) |  |
| T4 | 64 (18.9%) | 42 (21.0%) | 22 (15.9%) |  |
| Pathological N stage |  |  |  | 0.385 |
| N0 | 175 (51.6%) | 97 (48.5%) | 78 (56.1%) |  |
| N1 | 116 (34.2%) | 73 (36.5%) | 43 (30.9%) |  |
| N2 | 48 (14.2%) | 30 (15.0%) | 18 (13.0%) |  |
| Pathological M stage |  |  |  | 0.647 |
| M0 | 317 (93.5%) | 186 (93.0%) | 131 (94.2%) |  |
| M1 | 22 (6.5%) | 14 (7.0%) | 8 (5.8%) |  |
| Tumor differentiations |  |  |  | **<0.001** |
| Poorly | 82 (24.2%) | 72 (36.0%) | 10 (7.2%) |  |
| Moderately | 246 (72.6%) | 123 (61.5%) | 123 (88.5%) |  |
| Well | 11 (3.2%) | 5 (2.5%) | 6 (4.3%) |  |
| Neoadjuvant therapy | 147 (43.4%) | 88 (44.0%) | 59 (42.4%) | 0.776 |
| Adjuvant therapy | 304 (89.7%) | 182 (91.0%) | 122 (87.8%) | 0.336 |
| Neutrophil count (x10^9^/L) | 3.6 (2.5-4.8) | 3.8 (2.6-4.8) | 3.4 (2.2-4.8) | 0.143 |
| Lymphocyte count (x10^9^/L) | 1.3 (0.9-1.7) | 1.3 (0.9-1.7) | 1.2 (0.9-1.6) | 0.285 |
| Hemoglobin (g/L) | 121.6 ± 19.1 | 126.8 ± 19.7 | 114.3 ± 15.5 | **<0.001** |
| Cholesterol (mmol/L) | 4.3 (3.6-5.0) | 4.2 (3.5-4.8) | 4.4 (3.8-5.2) | **0.004** |
| LDL (mmol/L) | 2.3 ± 0.8 | 2.3 ± 0.8 | 2.4 ± 0.8 | 0.168 |
| HDL (mmol/L) | 1.1 (0.9-1.4) | 1.0 (0.8-1.3) | 1.3 (1.0-1.6) | **<0.001** |
| Albumin (g/L) | 41.7 (38.2-44.7) | 41.5 (38.1-44.5) | 41.9 (38.3-44.7) | 0.797 |
| PNI | 48.0 (44.1-51.9) | 47.8 (44.0-52.0) | 48.8 (44.2-51.9) | 0.950 |
| SMI (cm^2^/m^2^) | 43.1 (38.0-48.3) | 46.2 (42.3-50.5) | 38.2 (34.1-41.5) | **<0.001** |
| SATI (cm^2^/m^2^) | 40.8 (28.3-53.2) | 34.7 (25.7-43.5) | 53.0 (41.3-70.5) | **<0.001** |
| VATI (cm^2^/m^2^) | 37.0 (22.6-53.1) | 41.7 (24.8-57.4) | 32.2 (19.1-44.6) | **0.001** |
| SMD (HU) | 35.6 (31.1-40.1) | 37.5 (33.4-41.6) | 31.8 (27.6-35.9) | **<0.001** |

Bold indicates that the relevant data has statistical signiﬁcance.

Abbreviations: BMI, body mass index; CA 19-9, carbohydrate antigen 19-9; CEA, carcinoembryonic antigen; LDL, low-density lipoprotein; HDL, high-density lipoprotein; PNI, prognostic nutritional index; SMI, skeletal muscle index; SATI, subcutaneous adipose tissue index; VATI, visceral adipose tissue index; SMD, skeletal muscle density.

Supplementary Table 3. Univariate analysis of baseline characteristics and overall survival in PDAC patients.

|  | HR (95% CI) | *P* value |
| --- | --- | --- |
| Age (years) | 1.019 (1.001-1.037) | **0.041** |
| Male sex | 1.061 (0.781-1.442) | 0.704 |
| BMI (kg/m^2^) |  |  |
| < 25 | Reference |  |
| ≥ 25 | 0.692 (0.449-1.067) | 0.096 |
| Hypertension | 0.908 (0.660-1.250) | 0.555 |
| Diabetes | 0.860 (0.599-1.234) | 0.413 |
| CEA >5 (ng/ml) | 1.718 (1.226-2.408) | **0.002** |
| CA19-9 >37 (U/L) | 1.538 (1.108-2.134) | **0.010** |
| Tumor location |  |  |
| Head | Reference |  |
| Body and tail | 0.585 (0.428-0.798) | **0.001** |
| Tumor size, cm | 1.123 (1.013-1.244) | **0.027** |
| Pathological T stage |  | 0.080 |
| T1 | Reference |  |
| T2 | 1.684 (1.028-2.756) | **0.038** |
| T3 | 1.674 (0.999-2.807) | 0.051 |
| T4 | 2.037 (1.181-3.514) | **0.011** |
| Pathological N stage |  |  |
| N0 | Reference |  |
| N1 | 1.588 (1.130-2.234) | **0.008** |
| N2 | 2.322 (1.547-3.485) | **<0.001** |
| Pathological M stage |  |  |
| M0 | Reference |  |
| M1 | 1.197 (0.665-2.155) | 0.549 |
| Tumor differentiations |  | 0.085 |
| Poorly | Reference |  |
| Moderately | 0.828 (0.589-1.164) | 0.278 |
| Well | 0.220 (0.053-0.925) | **0.036** |
| Neoadjuvant therapy | 1.156 (0.852-1.570) | 0.352 |
| Adjuvant therapy | 0.603 (0.384-0.949) | **0.029** |
| Neutrophil count (x10^9^/L) | 1.028 (0.960-1.102) | 0.426 |
| Lymphocyte count (x10^9^/L) | 0.590 (0.437-0.795) | **0.001** |
| Hemoglobin (g/L) | 0.984 (0.976-0.992) | **<0.001** |
| Cholesterol (mmol/L) | 0.966 (0.833-1.120) | 0.647 |
| LDL (mmol/L) | 0.856 (0.707-1.037) | 0.113 |
| HDL (mmol/L) | 1.063 (0.721-1.567) | 0.759 |
| Albumin (g/L) | 0.920 (0.890-0.950) | **<0.001** |
| PNI | 0.932 (0.909-0.956) | **<0.001** |
| SMI (cm^2^/m^2^) | 0.948 (0.928-0.969) | **<0.001** |
| SATI (cm^2^/m^2^) | 0.989 (0.982-0.997) | **0.008** |
| VATI (cm^2^/m^2^) | 0.985 (0.977-0.993) | **<0.001** |
| SMD（HU） | 0.990 (0.970-1.023) | 0.375 |

Bold indicates that the relevant data has statistical signiﬁcance.

Abbreviations: BMI, body mass index; CA 19-9, carbohydrate antigen 19-9; CEA, carcinoembryonic antigen; LDL, low-density lipoprotein; HDL, high-density lipoprotein; PNI, prognostic nutritional index; SMI, skeletal muscle index; SATI, subcutaneous adipose tissue index; VATI, visceral adipose tissue index; SMD, skeletal muscle density.

Supplementary Table 4. The sex-specific cutoff values for each body composition parameter and their combinations with PNI in the training cohort.

|  | Male |  |  | Female |  |  |
| --- | --- | --- | --- | --- | --- | --- |
|  | Q1/Q2 | Q2/Q3 | Q3/Q4 | Q1/Q2 | Q2/Q3 | Q3/Q4 |
| SMI | 42.2 | 46.2 | 50.5 | 34.1 | 38.0 | 41.5 |
| SATI | 25.8 | 34.7 | 43.5 | 41.5 | 53.0 | 71.0 |
| VATI | 25.0 | 41.2 | 57.3 | 19.0 | 32.5 | 44.7 |
| SMD | 33.4 | 37.6 | 41.5 | 27.3 | 31.7 | 35.8 |
| SMI×PNI | 1900 | 2235 | 2560 | 1610 | 1820 | 2050 |
| SATI×PNI | 1203 | 1670 | 2125 | 1910 | 2530 | 3316 |
| VATI×PNI | 1130 | 2010 | 2870 | 850 | 1528 | 2287 |
| SMD×PNI | 1520 | 1798 | 2020 | 1285 | 1535 | 1820 |

Abbreviations: SMI, skeletal muscle index; SATI, subcutaneous adipose tissue index; VATI, visceral adipose tissue index; SMD, skeletal muscle density; PNI, prognostic nutritional index.

Additional References

S1. Xu, Z.-H., Wang, W.-Q., Lou, W.-H. & Liu, L. Insight of pancreatic cancer: recommendations for improving its therapeutic efficacy in the next decade. Journal of Pancreatology 5, 58–68 (2022).

S2. Park, W., Chawla, A. & O’Reilly, E. M. Pancreatic Cancer: A Review. JAMA 326, 851–862 (2021).

S3. Sabel, M. S. et al. Sarcopenia as a prognostic factor among patients with stage III melanoma. Ann Surg Oncol 18, 3579–3585 (2011).

S4. Li, J.-H. et al. Relationships of body composition and adipocytokines with outcomes in metastatic castration-resistant prostate cancer patients receiving docetaxel chemotherapy. Asian J Androl 25, 520527 (2023).

S5. Buzby, G. P. et al. Study protocol: a randomized clinical trial of total parenteral nutrition in malnourished surgical patients. Am J Clin Nutr 47, 366–381 (1988).

S6. Déniz, C. et al. Preoperative Omega-6/Omega-3 Fatty Acid Ratio Could Predict Postoperative Outcomes in Patients with Surgically Resected Non-Small-Cell Lung Cancer. Curr Oncol 29, 7086–7098 (2022).

S7. Migita, K. et al. The prognostic nutritional index predicts long-term outcomes of gastric cancer patients independent of tumor stage. Ann Surg Oncol 20, 2647–2654 (2013).

S8. Zhang, P. et al. Development of serum parameters panels for the early detection of pancreatic cancer. Int J Cancer 134, 2646–2655 (2014).

S9. Błogowski, W. et al. Selected cytokines in patients with pancreatic cancer: a preliminary report. PLoS One 9, e97613 (2014).

S10. Miller, K. D. et al. Visceral abdominal-fat accumulation associated with use of indinavir. Lancet 351, 871–875 (1998).

S11. von Meyenfeldt, M. Cancer-associated malnutrition: an introduction. Eur J Oncol Nurs 9 Suppl 2, S35-38 (2005).
